# Immunogenicity and safety of primary and booster vaccination with 2 investigational formulations of diphtheria, tetanus and *Haemophilus influenzae* type b antigens in a hexavalent DTPa-HBV-IPV/Hib combination vaccine in comparison with the licensed *Infanrix hexa*

**DOI:** 10.1080/21645515.2017.1294294

**Published:** 2017-03-24

**Authors:** Timo Vesikari, Luis Rivera, Tiina Korhonen, Anitta Ahonen, Brigitte Cheuvart, Marjan Hezareh, Winnie Janssens, Narcisa Mesaros

**Affiliations:** a Vaccine Research Center, University of Tampere, Tampere, Finland; b Hospital Maternidad Nuestra Señora de la Altagracia Santo Domingo, Santo Domingo, Dominican Republic; c University of Tampere, Tampere Vaccine Research Clinic, Tampere, Finland; d Vaccine Research Center, University of Tampere, Järvenpää Vaccine Clinic, Järvenpää, Finland; e GSK, Wavre, Belgium; f Chiltern International c/o GSK, Wavre, Belgium; g GSK, Wavre, Belgium

**Keywords:** acellular pertussis, DTPa-HBV-IPV/Hib, diphtheria, hepatitis B, *Haemophilus influenzae* type b, immunogenicity, infants, poliovirus, safety, tetanus

## Abstract

Safety and immunogenicity of 2 investigational formulations of diphtheria, tetanus and *Haemophilus influenzae* type b antigens of the combined diphtheria-tetanus-acellular pertussis-hepatitis B-inactivated poliomyelitis-Hib vaccine (DTPa-HBV-IPV/Hib) were evaluated in a Primary (NCT01248884) and a Booster vaccination (NCT01453998) study.

In the Primary study, 721 healthy infants (randomized 1:1:1) received 3 doses of DTPa-HBV-IPV/Hib formulation A (D_A_T_A_Pa-HBV-IPV/Hib), or B (D_B_T_B_Pa-HBV-IPV/Hib) or the licensed DTPa-HBV-IPV/Hib vaccine (*Infanrix hexa*, GSK; control group) at 2, 3, 4 months of age. Infants were planned to receive a booster dose at 12–15 months of age with the same formulation received in the Primary study; however, following high incidence of fever associated with the investigational formulations in the Primary study, the Booster study protocol was amended and all infants yet to receive a booster dose (N = 385) received the licensed vaccine.

In the Primary study, non-inferiority of 3-dose vaccination with investigational formulations compared with the licensed vaccine was not demonstrated due to anti-pertactin failing to meet the non-inferiority criterion. Post-primary vaccination, most infants had seroprotective levels of anti-diphtheria (100% of infants), anti-tetanus antigens (100%), against hepatitis B (≥ 97.5% across groups), polyribosyl-ribitol-phosphate (≥ 88.0%) and poliovirus types 1–3 (≥ 90.5%). Seropositivity rates for each pertussis antigen were 100% in all groups.

Higher incidence of fever (> 38°C) was reported in infants receiving the investigational formulations (Primary study: 75.0% [A] and 72.1% [B] vs 58.8% [control]; Booster study, before amendment: 49.4% and 46.6% vs 37.4%, respectively).

The development of the investigational formulations was not further pursued.

## Introduction

Combining multiple antigens into a single vaccine has several potential advantages including simplified administration, higher vaccine coverage, reduction in vaccination costs and number of visits, and minimized risk of administration errors and missed doses.^1,2^

A combined hexavalent diphtheria (D), tetanus (T), acellular pertussis (Pa), hepatitis B (HBV), inactivated poliomyelitis (IPV), and *Haemophilus influenzae* type b (Hib) vaccine (DTPa-HBV-IPV/Hib; *Infanrix hexa*, GSK) was first authorized for use in 2000.^3^ DTPa-HBV-IPV/Hib is indicated for primary vaccination as a 2- or 3-dose primary vaccination course in infants, followed by a booster vaccination with an interval of at least 6 months between the last dose of primary vaccination and the booster dose.

The currently licensed formulation of DTPa-HBV-IPV/Hib contains D and T antigens from Novartis Vaccines and Diagnostics, while the other antigens are manufactured in-house. In response to an increasing demand for DTPa-based vaccines and to increase supply flexibility, alternative formulations of diphtheria and tetanus antigens for use in DTPa combination vaccines have been developed and tested in pre-clinical settings and were proposed to progress in clinical evaluation. The historical manufacturing facilities could potentially not be able to face the increasing requirements for DTPa combination vaccines. Increasing the manufacturing capability would overcome this rising demand and help controlling the whole manufacturing process. Two DTPa-HBV-IPV/Hib formulations containing new diphtheria and tetanus antigens (D_A_T_A_Pa-HBV-IPV/Hib and D_B_T_B_Pa-HBV-IPV/Hib, formulations A and B, respectively), detoxified and adsorbed on aluminum hydroxide used as an adjuvant following 2 different processes (A and B), were chosen for clinical development. Additionally, in both investigational formulations, the Hib antigen was conjugated to the investigational tetanus toxoid (TT). We aimed to evaluate the immunogenicity and safety of the 2 new formulations administered as a primary 3-dose vaccination to infants at 2, 3 and 4 months of age (Primary vaccination study) and as a booster dose at 12–15 months of age (Booster study). All infants were co-administered with a 13-valent pneumococcal conjugate vaccine (PCV13; *Prevenar 13*™, Pfizer Inc.). The licensed formulation of DTPa-HBV-IPV/Hib vaccine was used as a benchmark to investigate non-inferiority of the immune response to all vaccine antigens.

## Results

### Study participants

**Figure 1.**
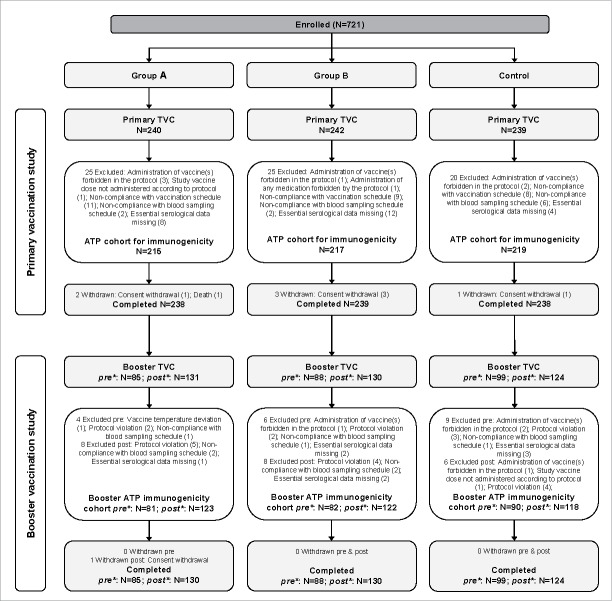
Flow of participants in the Primary and Booster vaccination studies. N, number of participants, TVC, total vaccinated cohort; ATP, according to protocol; group A/group B, infants who received the new formulations A or B of DTPa-HBV-IPV/Hib + PCV13 as a primary vaccination at 2, 3, 4 months of age and a booster dose with the same vaccine at 12–15 months of age (after protocol amendment, both groups received the licensed DTPa-HBV-IPV/Hib and PCV13 as booster); Control, infants who received the licensed DTPa-HBV-IPV/Hib + PCV13 as a primary vaccination at 2, 3, 4 months of age and a booster dose at 12–15 months of age; pre*, before protocol amendment; post*, after protocol amendment.

In the Primary study, a total of 721 infants, 456 infants from Finland and 265 infants from Dominican Republic, were enrolled and included in the total vaccinated cohort (TVC) (240 received formulation A [group A], 242 received formulation B [group B] and 239 received the licensed vaccine [control group]); of those, 651 (215 in group A, 217 in group B and 219 in the control group) were included in the according-to-protocol (ATP) cohort for immunogenicity (ATP-I) (Fig. 1). Screen failures were not recorded for the primary study.

In total, 657 infants (409 from Finland and 248 from Dominican Republic) were enrolled in the Booster study. The remaining 64 screened participants were not enrolled in the study due to withdrawal of consent (31), non-eligibility (14), lost to follow up (8), moving from the study area (7) or non-willingness for blood sampling (1). One infant had died (serious adverse event [SAE] described in *Safety – Primary study* section) and 2 did not participate because of an adverse event (AE; acute disease at enrolment and foot erythema). Initially, 85, 88 and 99 infants were enrolled in groups A, B and control, respectively, to receive a booster of the formulation as in the primary study; of these, 81 (group A), 82 (group B) and 90 (control group) were included in the ATP-I. Following a high incidence of fever observed in infants who received the investigational formulations in the Primary study (see *Safety* section of the Results), the study protocol was amended and all infants (N = 385) who were still to receive the booster dose, received the licensed formulation as booster. After the protocol amendment, 131, 130 and 124 infants were included in groups A, B and control, respectively; of these, 123 (group A), 122 (group B) and 118 (control group) were included in ATP-I (Table 1).

**Table 1.** Summary of demographic characteristics (total vaccinated cohorts).

	TVC		
	Group A (N = 240)	Group B (N = 242)	Control group (N = 239)
**Primary study**			
Age at dose 1 (we)			
Mean	9.7	9.8	9.7
Range (min–max)	8–12	8–12	8–12
Female/male, %	49.6/50.4	57.0/43.0	41.4/58.6
Ancestry, n (%)			
White Caucasian	144 (60.0)	148 (61.2)	140 (58.6)
Other	96 (40.0)	94 (38.8)	99 (41.4)
**Booster study** (Before protocol amendment)	Group A (N = 85)	Group B (N = 88)	Control group (N = 99)
Age at booster dose (mo)			
Mean	12.9	13.0	13.0
Range (min–max)	12–15	12–15	12–15
Female/male, %	55.3/44.7	55.7/44.3	38.4/61.6
Ancestry, n (%)			
White Caucasian	79 (92.9)	83 (94.3)	91 (91.9)
Other	6 (7.1)	5 (5.7)	8 (8.1)
			
**Booster study** (After protocol amendment)	Group A (N = 131)	Group B (N = 130)	Control group (N = 124)
Age at booster dose (mo)			
Mean	14.1	13.9	14.0
Range (min-–max)	12–16	12–15	12–15
Female/male, %	46.6/53.4	58.5/41.5	42.7/57.3
Ancestry, n (%)			
White Caucasian	49 (37.4)	46 (35.4)	39 (31.5)
Other	82 (62.6)	84 (64.6)	85 (68.5)

N, number of participants; n (%), number (percentage) of participants in a given category; TVC, total vaccinated cohort; we, weeks; mo, months; min, minimum; max, maximum. In the Primary and Booster studies (before protocol amendment) groups A and B received the investigational formulations A and B of DTPa-HBV-IPV/Hib + PCV13 at 2, 3 and 4 months of age and as a booster dose at 12–15 months of age; the control group received the licensed DTPa-HBV-IPV/Hib + PCV13 vaccines at 12–15 months of age. After protocol amendment of the Booster study, all 3 groups received the licensed DTPa-HBV-IPV/Hib + PCV13 vaccines.

### Immunogenicity

#### Primary study

The non-inferiority of the immunogenicity of the investigational DTPa-HBV-IPV/Hib formulations compared with the licensed vaccine was assessed in terms of seroprotection rates to diphtheria and tetanus antigens, hepatitis B surface antigens (HBsAg), and polyribosyl-ribitol-phosphate antigens (PRP, a polysaccharide component of *Haemophilus influenzae* bacterium capsule associated with virulence), and in terms of antibody geometric mean concentrations (GMCs) for pertussis antigens one month after the third vaccine dose. The non-inferiority of the investigational D_A_T_A_Pa-HBV-IPV/Hib and D_B_T_B_Pa-HBV-IPV/Hib formulations to the licensed vaccine was not demonstrated as the upper limits (ULs) of the 97.5% confidence intervals (CIs) of anti-pertactin (PRN) GMC ratio (control group/investigational formulation) exceeded the pre-defined limit of 1.5 for both formulations (A: 1.54 and B: 1.84); of note, the non-inferiority criteria were met for all other antigens (Table 2).

**Table 2.** Group differences in seroprotection/seropositivity rates and adjusted GMC ratio one month post-dose 3 in the Primary vaccination study (ATP cohort for immunogenicity).

		Control	Group A	Difference in percentage (control group minus group A)	Group B	Difference in percentage (control group minus group B)
Antibody		n (%)	n (%)	% (97.5% CI)	n (%)	% (97.5% CI)
Anti-D (0.1 IU/mL)		219 (100)	214 (100)	0.00 (−2.25–2.30)	217 (100)	0.00 (−2.25–2.27)
Anti-T (0.1 IU/mL)		219 (100)	214 (100)	0.00 (−2.25–2.30)	217 (100)	0.00 (−2.25–2.27)
Anti- HBs	10 mIU/mL, in-house ELISA	205 (98.1)	197 (97.5)	0.56 (−3.27–4.63)	203 (99.0)	−0.94 (−4.57–2.36)
	10 mIU/mL, CLIA adjusted	203 (97.1)	197 (97.5)	−0.40 (−4.62–3.80)	201 (98.0)	−0.92 (−5.07–3.02)
Anti-PRP (0.15 *µ*g/mL)		193 (88.5)	197 (92.1)	−3.52 (−10.19–3.00)	190 (88.0)	0.57 (−6.53–7.70)
		**Adjusted GMC**		**Adjusted GMC ratio (control group/group A)**	**Adjusted GMC**	**Adjusted GMC ratio (control group/group B)**
**Pertussis antigens**		**Control**	**Group A**	**Value (97.5% CI)**	**Group B**	**Value (97.5% C)**
Anti-PT (EU/mL)		73.9	58.5	1.26 (1.11–1.44)	59.0	1.25 (1.10–1.43)
Anti-FHA (EU/mL)		207.6	193.0	1.08 (0.94–1.23)	166.6	1.25 (1.09–1.42)
Anti-PRN (EU/mL)		105.6	79.5	1.33 (1.14–1.54)	66.7	1.58 (1.37–1.84)

ATP, according-to-protocol; n (%), number (percentage) of participants with antibody concentration above the specified cut-off; CI, confidence interval; D, diphtheria; T, tetanus; PRP, polyribosyl-ribitol phosphate; HBs, hepatitis B; PT, pertussis toxoid; FHA, filamentous hemagglutinin; PRN, pertactin; CLIA, ChemiLuminescence ImmunoAssay; ELISA, enzyme-linked immunosorbent assay; EU/ml, ELISA units per milliliter; IU/ml, international units per milliliter. Adjusted GMC ratio, geometric mean antibody concentration adjusted for baseline concentration. In the Primary study, groups A and B received the investigational formulations A and B of DTPa-HBV-IPV/Hib + PCV13 at 2, 3 and 4 months of age; control group received the licensed DTPa-HBV-IPV/Hib+PCV13 vaccine at 2, 3 and 4 months of age.

One month post-dose 3, seroprotective/seropositive concentrations of antibodies against diphtheria, tetanus and all pertussis antigens were observed in all infants in the 3 groups, and at least 97.5% of infants in all groups had seroprotective levels of anti-HBs antibodies, at least 88.0% of infants had anti-PRP antibody concentrations ≥ 0.15 *µ*g/mL, and at least 97.4%, 90.5% and 97.9% of infants had seroprotective titers of antibodies against poliovirus types 1, 2 and 3 across the 3 groups, respectively (Table 3). Seropositivity rates for each pertussis antigen were 100% in all groups.

**Table 3.** Seroprotection/seropositivity rates and GMCs/GMTs before and one month post-dose 3 in the Primary and Booster vaccination studies (ATP-I cohorts).

		Primary Vaccination					Booster Vaccination before amendment				Booster Vaccination after amendment			
Antibody	Groups	Time point	%SP	95% CI	GMC/GMT	95% CI	%SP	95% CI	GMC/GMT	95% CI	%SP	95% CI	GMC/GMT	95% CI
Anti-D	A	Pre	77.0	70.5–82.6	0.292	0.247–0.347	96.3	89.6–99.2	0.357	0.305–0.419	87.0	79.7–92.4	0.247	0.213–0.287
(≥ 0.1 IU/mL)		Post	100	98.3–100	1.499	1.367–1.644	100	95.5–100	5.652	4.985–6.408	100	97.0–100	6.327	5.698–7.025
	B	Pre	78.6	72.4–83.9	0.281	0.238–0.332	97.6	91.5–99.7	0.445	0.381–0.520	94.2	88.4–97.6	0.278	0.244–0.317
		Post	100	98.3–100	1.704	1.564–1.856	100	95.6–100	5.494	4.891–6.171	100	97.0–100	5.452	4.956–5.998
	Control	Pre	75.8	69.5–81.4	0.290	0.245–0.343	94.4	87.4–98.2	0.401	0.343–0.468	88.9	81.7–93.9	0.304	0.258–0.360
		Post	100	98.3–100	1.839	1.686–2.005	100	96.0–100	6.772	5.897–7.777	100	96.9–100	7.192	6.419–8.059
Anti-T	A	Pre	98.5	95.7–99.7	0.936	0.832–1.053	93.8	86.2–98.0	0.358	0.301–0.427	94.3	88.6–97.7	0.364	0.313–0.422
(≥ 0.1 IU/mL)		Post	100	98.3–100	1.761	1.624–1.910	100	95.5–100	5.015	4.341–5.794	100	97.0–100	5.986	5.204–6.885
	B	Pre	98.1	95.2–99.5	0.920	0.822–1.029	95.1	88.0–98.7	0.362	0.306–0.428	94.2	88.4–97.6	0.332	0.289–0.380
		Post	100	98.3–100	1.726	1.597–1.865	100	95.6–100	5.034	4.366–5.803	100	97.0–100	5.316	4.716–5.992
	Control	Pre	99.1	96.6–99.9	0.907	0.812–1.013	95.5	88.9–98.8	0.394	0.337–0.459	94.9	89.2–98.1	0.331	0.285–0.383
		Post	100	98.3–100	1.947	1.818–2.085	100	96.0–100	5.571	4.869–6.374	100	96.9–100	5.993	5.222–6.878
Anti-PRP	A	Pre					50.6	39.3–61.9	0.173	0.138–0.216	74.8	66.2–82.2	0.328	0.262–0.409
(≥ 0.15 *µ*g/mL)		Post	92.1	87.6–95.3	0.951	0.793–1.142	100	95.5–100	12.765	9.300–17.520	99.2	95.6–100	21.462	16.65–27.664
	B	Pre					54.9	43.5–65.9	0.175	0.142–0.216	64.5	55.2–73	0.288	0.227–0.365
		Post	88.0	82.9–92.0	0.730	0.606–0.880	100	95.6–100	15.904	11.723–21.576	100	97.0–100	15.903	12.132–20.848
	Control	Pre					58.4	47.5–68.8	0.236	0.182–0.307	69.2	60.0–77.4	0.334	0.254–0.439
		Post	88.5	83.5–92.4	1.082	0.884–1.324	100	96–100	17.099	12.966–22.55	99.2	95.4–100	17.429	13.429–22.620
Anti-PT	A	Pre	16.6	11.7–22.5	3.3	3.0–3.6	85.0	75.3–92.0	10.5	8.8–12.6	77.2	68.8–84.3	8.3	7.2–9.7
(≥ 5 EU/mL)		Post	100	98.3–100	57.7	52.9–62.9	100	95.4–100	76.1	66.1–87.6	100	96.9–100	92.4	80.6–106
	B	Pre	18.1	13.1–24	3.4	3.1–3.7	81.3	71.0–89.1	9.5	7.9–11.4	77.7	69.2–84.8	7.9	6.8–9.1
		Post	100	98.3–100	57.5	53.1–62.4	100	95.5–100	74.3	62.6–88.1	100	97.0–100	93.6	83.1–105.5
	Control	Pre	14.8	10.3–20.3	3.1	2.9–3.4	89.7	81.3–95.2	12.7	10.8–15.0	82.9	74.8–89.2	9.9	8.5–11.5
		Post	100	98.3–100	73.2	67.7–79.2	100	95.9–100	96.0	83.5–110.3	100	96.9–100	132.6	114.9–153.0
Anti-FHA	A	Pre	80.6	74.4–85.9	10.6	9.3–12.2	100	95.5–100	41.7	35.4–49.2	99.2	95.6–100	37.6	32.5–43.4
(≥ 5 EU/mL)		Post	100	98.3–100	192.4	175–211.4	100	95.5–100	393.7	346.4–447.6	100	97.0–100	467.3	417.3–523.3
	B	Pre	76.7	70.4–82.2	9.7	8.5–11.1	98.8	93.4–100	36.9	31.5–43.3	99.2	95.4–100	34.0	28.7–40.4
		Post	100	98.3–100	165.5	151.5–180.7	100	95.6–100	372.4	332.7–416.7	100	97.0–100	446.2	402.3–494.9
	Control	Pre	75.0	68.5–80.7	9.1	8.0–10.4	100	95.9–100	47.1	40.3–55.1	100	96.9–100	45.7	38.8–53.9
		Post	100	98.3–100	210.6	194.1–228.6	100	96.0–100	423.0	368.1–485.9	100	96.9–100	582.9	517.1–657.1
Anti-PRN	A	Pre	42.0	35.1–49.2	5.1	4.5–5.9	84.0	74.1–91.2	12.8	10.4–15.7	79.7	71.5–86.4	11.6	9.7–13.9
(≥ 5 EU/mL)		Post	100	98.3–100	76.6	68.1–86.3	100	95.5–100	213.0	178.1–254.7	100	97.0–100	253.2	216.9–295.6
	B	Pre	42.4	35.6–49.4	4.9	4.3–5.5	85.2	75.6–92.1	10.8	8.9–13.1	76.0	67.4–83.3	9.7	8.1–11.7
		Post	100	98.3–100	65.7	58.9–73.3	100	95.5–100	180.0	154.2–210.1	100	97.0–100	181.0	154.8–211.7
	Control	Pre	36.2	29.7–43.1	4.9	4.3–5.7	93.3	85.9–97.5	18.2	15.0–22.1	89.7	82.8–94.6	15.6	13.0–18.7
		Post	100	98.3–100	106.6	96.6–117.8	100	95.9–100	372.9	309.3–449.5	100	96.9–100	401.1	342.2–470.0
Anti-HBs	A	Pre					91.9	83.2–97.0	130.3	91.2–186.0	90.0	83.2–94.7	94.9	72.2–124.8
(≥ 10 mIU/mL)		Post	97.5	94.3–99.2	639.5	523.6–781.2	98.7	93.1–100	2233.3	1479.7–3370.8	98.4	94.2–99.8	2229.3	1625.5–3057.5
	B	Pre					93.7	85.8–97.9	124.4	89.5–173.0	84.0	76.2–90.1	61.8	45.7–83.5
		Post	99.0	96.5–99.9	602.6	492.1–737.9	98.7	93.1–100	2026.3	1389.4–2955.2	98.3	93.9–99.8	1729.8	1240.6–2411.9
	Control	Pre					92.9	85.1–97.3	166.4	112.8–245.5	92.2	85.7–96.4	125.9	94.6–167.7
		Post	98.1	95.2–99.5	799.0	662.2–964.0	100	95.7–100	2685.7	1868.8–3859.7	99.1	95.3–100	3711.4	2729.7–5046.1
Anti-poliovirus type 1 (≥ 8 ED_50_)	A	Pre	63.9	56.7–70.7	13.5	11.5–16.0	72.5	60.4–82.5	18.2	13.7–24.1	89.6	82.2–94.7	53.5	39.6–72.4
		Post	97.4	94.1–99.2	110.0	88.6–136.5	98.7	93.0–100	572.9	435.5–753.6	100	96.7–100	1121.0	904.2–1389.8
	B	Pre	67.0	60.1–73.4	13.3	11.4–15.5	73.0	61.4–82.6	17.8	13.5–23.5	86.7	78.6–92.5	50.7	37.2–69.2
		Post	97.5	94.2–99.2	94.6	77.2–116.0	100	95.0–100	558.3	422–738.8	100	96.6–100	1099.6	905.2–1335.8
	Control	Pre	58.4	51.3–65.3	13.2	11.1–15.8	78.9	67.6–87.7	22.4	16.8–29.9	92.9	86.0–97.1	70.8	52.4–95.8
		Post	97.5	94.4–99.2	143.8	117.7–175.7	100	95.8–100	902.1	698.4–1165.0	99.0	94.7–100	1386.2	1091.8–1760.0
Anti-poliovirus type 2 (≥ 8 ED_50_)	A	Pre	67.0	59.6–73.9	16.0	13.3–19.3	55.1	42.6–67.1	12.7	9.5–16.9	86.7	77.9–92.9	76.6	50.3–116.7
		Post	90.5	85.4–94.3	72.0	57.3–90.4	100	94.2–100	629.7	452.6–876.1	100	96.2–100	1485.3	1182.4–1865.8
	B	Pre	71.9	64.8–78.2	18.4	15.2–22.2	61.1	48.9–72.4	17.1	12.3–23.8	87.1	78.5–93.2	55.0	38.2–79.3
		Post	94.8	90.6–97.5	68.5	55.3–84.9	98.4	91.2–100	668.7	489.9–912.7	99.0	94.4–100	1215.6	973.8–1517.4
	Control	Pre	70.4	63.3–76.8	18.8	15.5–22.8	63.8	51.3–75.0	16.6	12.0–22.8	87.1	78.0–93.4	82.7	55.6–122.9
		Post	93.4	89.0–96.4	81.0	65.1–101.0	100	95.3–100	1184.9	901.1–1558.1	100	95.8–100	1537.2	1191.0–1984.1
Anti-poliovirus type 3 (≥ 8 ED_50_)	A	Pre	51.8	44.5–59.0	13.0	10.7–15.8	69.9	58.0–80.1	24.7	17.1–35.8	88.9	81.4–−4.1	67.8	47.3–97.2
		Post	97.9	94.8–99.4	179.4	141.2–227.9	100	94.4–100	1147.5	846.2–1556.0	100	96.8–100	1851.2	1473.2–2326.1
	B	Pre	51.2	44.2–58.2	12.5	10.4–15.0	58.9	46.8–70.3	16.8	12.0–23.5	90.8	83.8–95.5	73.8	52.2–104.2
		Post	97.9	94.8–99.4	159.6	126.9–200.8	100	94.3–100	614.0	453.9–830.6	100	96.5–100	1960.4	1574.0–2441.5
	Control	Pre	53.2	46.1–60.2	12.6	10.5–15.1	76.3	65.4–85.1	26.6	19.2–36.9	87.5	79.6–93.2	93.9	64.4–136.8
		Post	98.9	96.2–99.9	221.7	176.1–279.2	97.3	90.5–99.7	1120.7	793.0–1583.9	100	96.3–100	2376.4	1874.2–3013.2

ATP-I, according-to-protocol (cohort for) immunogenicity; %SP, percentage of seroprotected/seropositive infants; GMC/GMT, geometric mean antibody concentration/titer; 95% CI, 95% confidence interval; Pre, pre-primary/booster vaccination; Post, post-dose 3/booster vaccination; D, diphtheria; T, tetanus; PRP, polyribosyl-ribitol phosphate; PT, pertussis toxoid; FHA, filamentous hemagglutinin; PRN, pertactin, HBs, hepatitis B; EU/ml, ELISA units per milliliter; IU/ml, international units per milliliter; ED_50_, median effective dose. In the Primary and Booster studies (before protocol amendment), groups A and B received the investigational formulations A and B of DTPa-HBV-IPV/Hib + PCV13 at 2, 3 and 4 months of age and as a booster dose at 12–15 months of age; the control group received the licensed DTPa-HBV-IPV/Hib + PCV13 vaccines at 12–15 months of age. After protocol amendment of the Booster study, all 3 groups received the licensed DTPa-HBV-IPV/Hib + PCV13 vaccines.

All samples with anti- HBs antibody concentrations between 10–100 mIU/mL at one month after the primary vaccination by the in-house ELISA (considered overestimated), were retested with the commercial ChemiLuminescence ImmunoAssay (CLIA) with a cut-off defining seropositivity of 6.2 mIU/mL. Anti-HBs seroprotection was redefined as in-house ELISA concentration above 100 mIU/mL (considered valid) or CLIA concentration above 10 mIU/mL. CLIA was also used for the Booster study.

Vaccine response to PT, FHA and PRN was mounted in at least 97.1%, 96.9% and 91.0% of infants, respectively (Table 4).

**Table 4.** Vaccine response rate to anti-PT, anti-FHA and anti-PRN antibodies one month post-primary vaccination (ATP cohort for immunogenicity).

	% Vaccine response (95% CI)		
Antibody	Group A (N = 181)	Group B (N = 194)	Control (N = 198)
Anti-PT	98.0 (94.9–99.4)	97.1 (93.9–98.9)	99.0 (96.6–99.9)
Anti-FHA	96.9 (93.5–98.9)	97.6 (94.5–99.2)	98.1 (95.1–99.5)
Anti-PRN	91.0 (86.1–94.6)	93.3 (89.0–96.3)	94.3 (90.2–97.0)

ATP, according-to-protocol; %, percentage of infants with vaccine response; CI, confidence interval; PT, pertussis toxoid; FHA, filamentous hemagglutinin; PRN, pertactin; N, minimum number of infants with available results.

Groups A and B received the investigational formulations A and B of DTPa-HBV-IPV/Hib + PCV13 at 2, 3 and 4 months of age.

Control group received the licensed DTPa-HBV-IPV/Hib + PCV13 vaccines at 2, 3 and 4 months of age.

Vaccine response was defined as a post-dose 3 antibody concentration ≥ 5 ELISA units/mL (EU/mL) for initially seronegative infants, or an antibody concentration ≥ 1-fold the pre-vaccination antibody concentration for initially seropositive infants. Infants with antibody concentration < 5 EU/mL before vaccination were considered seronegative; infants with antibody concentration ≥ 5 EU/mL before vaccination were considered seropositive.

#### Booster study

The percentage of infants with anti-D, anti-T, anti-HBs, anti-PRP and anti-poliovirus types 1–3 antibody concentrations above the seroprotective cut-offs one month after booster vaccination was at least 97.3% before the protocol amendment, and at least 98.3% after the amendment. With respect to pertussis, higher GMCs were observed when the licensed vaccine was administered in the primary phase (Table 3).

### Safety

#### Primary study

**Figure 2.**
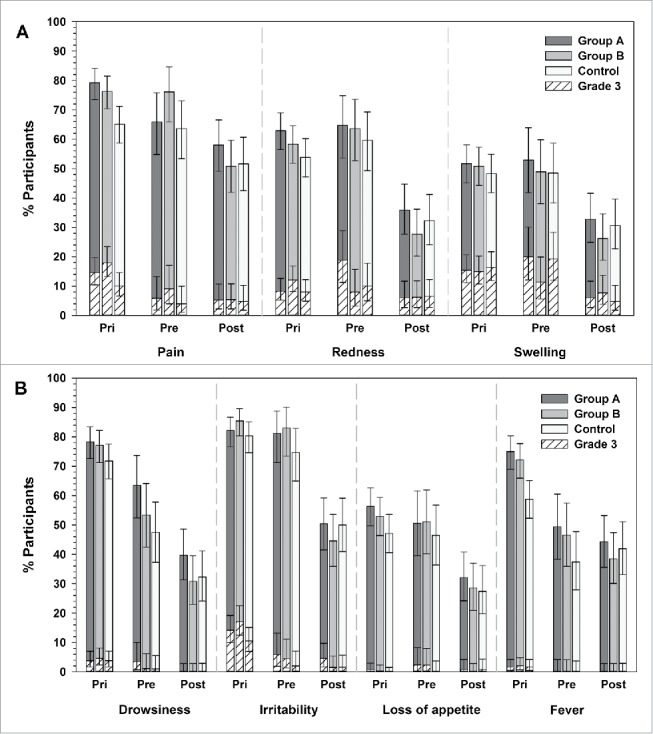
Incidence of solicited local (A) and general symptoms (B) in Primary (day 0–7) and Booster study (day 0–4) (total vaccinated cohorts). Group A/Group B, infants who received the new formulations A or B of DTPa-HBV-IPV/Hib + PCV13 as a primary vaccination at 2, 3, 4 months of age and a booster dose with the same vaccine at 12–15 months of age (after protocol amendment, both groups received the licensed DTPa-HBV-IPV/Hib + PCV13 as booster); Control, infants who received the licensed DTPa-HBV-IPV/Hib + PCV13 as a primary vaccination at 2, 3, 4 months of age and a booster dose at 12–15 months of age; Pri, primary vaccination; Pre, booster vaccination before protocol amendment; Post, booster vaccination after protocol amendment. The error bars indicate 95% confidence intervals.

Injection site pain was the most frequently reported solicited local symptom in the 3 groups, reported in 79.2%, 70.4% and 65.1% of infants in groups A, B and control, respectively; the most common grade 3 solicited local symptom was swelling, reported in 15.4% of infants in group A, 15.0% of infants in group B, and 16.4% of infants in the control group (Fig. 2A).

Irritability was the most common solicited general symptom in all 3 groups (group A: 82.1%, group B: 85.4%, control: 80.3%), and was also the most common grade 3 symptom (group A: 14.2%, group B: 17.1%, control: 10.5%) (Fig. 2B). The incidence of fever reported in infants who received the investigational formulations appeared higher compared with control (group A: 75.0%, group B: 72.1%, control: 58.8%). The incidence of fever was in majority considered by the investigators to be related to vaccination and causally related fever incidence was 74.6% in group A, 70.0% in group B and 58.0% in the control group. Grade 3 fever (> 39.0°C axillary temperature) was reported for 1.7% of infants in group A and the control group, and for 2.1% of infants in group B (Fig. 2B).

During the 31-day post-vaccination period, at least one unsolicited adverse event was reported for 63.8%, 68.2%, and 66.5% of infants in groups A, B, and control, respectively; grade 3 unsolicited AEs were reported for 7.1%, 8.7% and 6.7% of infants and unsolicited AEs with a causal relationship to vaccination, for 21.7%, 21.1% and 23.0% of infants in these groups, respectively.

Twenty-one SAEs were reported for 18 infants (9, 5, and 4 infants in groups A, B and control, respectively). During the entire study period, one fatal SAE was reported in the Dominican Republic in group A 17 d post-dose 1, the reason being asphyxia and interstitial lung disease. None of the SAEs were considered by the investigator to be potentially related to vaccination.

### Booster study

#### Before protocol amendment

The most common solicited local symptom was injection site pain, reported for 65.9%, 76.1% and 63.6% of infants in groups A, B, and control, respectively; the most common grade 3 solicited local symptom was swelling, reported in 20.0% of infants in group A, 11.4% of infants in group B, and 19.2% of infants in the control group (Fig. 2A).

The incidence of solicited general symptoms ranged from 49.4%–81.2% in group A, from 46.6%–83.0% in group B, and from 37.4%–74.7% in the control group. Irritability was the most common solicited general symptom in all 3 groups (group A: 81.2%, group B: 83.0%, control: 74.7%), and was also the most common grade 3 symptom (group A: 5.9%, group B: 4.5%, control: 2.0%) (Fig. 2B). The incidence of fever appeared higher in groups A (49.4%) and B (46.6%) compared to control (37.4%); grade 3 fever (> 39.0°C axillary temperature) was not reported (Fig. 2B). The incidence of fever was in majority considered by the investigators to be related to vaccination and causally related incidence was 49.4% in group A, 45.5% in group B and 36.4% in the control group.

The incidence of unsolicited AEs reported up to day 31 following vaccination was similar in infants who received the investigational formulations or the licensed vaccine as booster (group A: 49.4%, group B: 44.3%, control: 50.5%). No SAEs were reported before protocol amendment.

#### After protocol amendment

The most common solicited local symptom in all groups was injection site pain (58.0% in group A, 50.8% in group B and 51.6% in control group). The most common grade 3 solicited local symptoms were redness (group A: 6.1%, group B: 6.2%, control: 6.5%) and swelling (group A: 6.1%, group B: 7.7%, control: 4.8%).

The most common general symptom in all groups was irritability reported in 50.4%, 44.6% and 50.0% of infants from group A, group B and control, respectively, and was also the most common grade 3 general symptom (group A: 4.6%, group B: 1.5%, control: 1.6%).

The incidence of fever was always considered by the investigators to be related to vaccination and appeared similar in all groups (group A: 44.3%, group B: 38.5%, control: 41.9%, respectively); grade 3 fever was not reported (Fig. 2B).

Three SAEs were reported after the amendment, 2 cases of pneumonia and a case of dehydration (all in group B); none were considered by the investigator to be related to vaccination and all recovered before the study end.

### Discussion

The immunogenicity of the investigational DTPa-HBV-IPV/Hib formulations administered to infants as a 3-dose primary vaccination was inferior to the licensed DTPa-HBV-IPV/Hib vaccine and the reactogenicity was higher.

In the Primary vaccination study reported in this manuscript, the incidence of fever following 3 doses of the investigational DTPa-HBV-IPV/Hib formulations was much higher (group A: 75.0%, group B: 72.1%), and was likely either due to the new formulations of the diphtheria and tetanus antigens or to the PRP that was conjugated to the investigational tetanus antigen. Nevertheless, the incidence of grade 3 fever reported for the investigational vaccine formulation post-primary vaccination was low (1.7%–2.1% across the groups), similar to previously reported for the investigational DTaP5-IPV-Hib-HepB formulation (2%).^4^

Altogether, the results of the Primary and Booster vaccination studies presented in this manuscript indicate that the DTPa-HBV-IPV/Hib vaccine formulations containing investigational diphtheria and tetanus antigens are associated with a lower immunogenicity and a higher reactogenicity compared to the licensed DTPa-HBV-IPV/Hib vaccine. The incidence of SAEs was low, consistent with the results of previous studies with the DTPa-HBV-IPV/Hib vaccine.^5,6^ This finding emphasizes the need to carry out head-to-head comparisons for any new compositions of hexavalent vaccine. This experience suggests that the formulation of a hexavalent vaccine is sensitive to changes in the vaccine components and new experimental combinations may be less immunogenic than the licensed *Infanrix hexa*.

In the light of waning immunity following vaccination with acellular pertussis vaccines, the need for a new generation of pertussis vaccines is recognized. Alternative formulations might be improved with the addition of more or improved antigens to the multicomponent vaccines, removal of antigens or adjuvant improvement, or use of DNA or attenuated, live bacterial vaccines.^7^

The studies had the following strengths: (1) enrolment from different settings (Finland and Dominican Republic) allowed assessing whether the observed effects were country specific; (2) the short vaccination schedule provided a worst case scenario for assessing seroprotection by the different vaccine formulations; (3) the drop-out rate was reasonably low for this type of study as approximately 85% of the infants completed both primary and booster studies. Another advantage of the study design was the availability of blood samples pre-vaccination, allowing anti-pertussis geometric mean titer (GMT) group comparisons while accounting for the pre-vaccination immunogenicity.

Potential limitations of the studies include the fact that inferential analysis linked to the primary objective in the Booster study could not be performed due to failure of the primary non-inferiority objective in the Primary vaccination study and due to the protocol amendment.

As the investigational formulations of the DTPa-HBV-IPV/Hib failed to demonstrate non-inferiority of the vaccine immunogenicity to the licensed formulation, and because of a higher incidence of fever associated with the investigational formulations compared to the licensed vaccine, the development of the investigational formulations was not further pursued.

## Patients and Methods

### Study design

The Primary vaccination study was a phase I/II double-blind, randomized, multicenter study conducted in 2 centers in the Dominican Republic and 14 centers in Finland between December 2010 and January 2012. Healthy infants were randomized (1:1:1) to receive 3 doses of 2 investigational DTPa-HBV-IPV/Hib formulations (A or B; group A and B, respectively) or the licensed DTPa-HBV-IPV/Hib formulation (control group) at 2, 3, 4 months of age; in addition, all infants received concomitant injections of PCV13.

Infants who received 3 doses of either formulation of DTPa-HBV-IPV/Hib vaccine (A, B or control [licensed vaccine]) in the Primary study were invited to participate in a follow-up, phase II, randomized, double-blind Booster vaccination study (October 2011–November 2012) to evaluate the response to the booster vaccination with DTPa-HBV-IPV/Hib received between 12 and 15 months of age. Infants participating in the Booster study retained the group allocation to which they were randomized in the Primary study. In addition, all infants received a concomitant booster dose of PCV13.

Initially, infants received a booster dose with the same vaccine formulations as in the Primary study; following a protocol amendment (see Results section), infants in all 3 groups received the licensed DTPa-HBV-IPV/Hib formulation as booster dose. The double-blinding regarding the vaccine received during the Primary study was maintained until the end of the Booster study.

Written informed consent was obtained for each infant from the parent or the legally acceptable representative (LAR). The study was conducted according to the Declaration of Helsinki, Good Clinical Practice, International Conference on Harmonisation (ICH) Harmonised Tripartite Guideline for clinical investigation of medicinal products in the pediatric population (ICH E11), and the Finnish and Dominican laws and regulations. The study protocol, the amendments, the informed consent, and all documents requiring pre-approval were reviewed and approved by an Institutional Review Board or an Independent Ethics Committee.

The studies are registered at www.clinicaltrials.gov (NCT01248884 and NCT01453998). A summary of each study protocol is available at http://www.gsk-clinicalstudyregister.com (GSK study ID: 113948 and 114843).

### Study objectives

#### Primary vaccination study

The primary objective of the study was to demonstrate non-inferiority of at least one of the 2 investigational DTPa-HBV-IPV/Hib formulations compared with the licensed formulation in terms of seroprotection rates to diphtheria, tetanus, HBsAg and PRP antigens, and in terms of antibody GMCs for pertussis antigens one month after the third dose.

The secondary objectives included the assessment of the immune response to the study vaccines in terms of: seroprotection/seropositivity and antibody concentrations or titers, one month after the third dose; immunological status toward diphtheria, tetanus, pertussis and polio antigens; seroprotection/seropositivity and antibody concentrations before the primary vaccination; and vaccine response to pertussis antigens one month after the third dose; the assessment of the safety and reactogenicity of the study vaccines.

#### Booster study

The primary objective was to demonstrate non-inferiority of at least one of the 2 investigational DTPa-HBV-IPV/Hib formulations compared with the licensed formulation in terms of seroprotection rates to diphtheria, tetanus, HBsAg, poliovirus types 1, 2 and 3 and PRP antigens, and in terms of GMCs for pertussis antigens one month after the booster dose.

Before protocol amendment, participants received a booster dose of the same vaccine they received as primary vaccination course. Following the protocol amendment, all the participants yet to receive the booster dose, received the licensed DTPa-HBV-IPV/Hib formulation. Accordingly, as only less than half of the participants could contribute to the initial study objectives leading to a study power lower than 50%, the inferential non-inferiority analysis was not performed, but separate descriptive analyses of the cohorts enrolled before and after the amendment were performed (see Statistical analysis section).

Secondary objectives included the assessment of the persistence of antibodies to all vaccine antigens at pre-booster; immune responses to the study vaccines in terms of seroprotection/seropositivity status and antibody concentrations or titers for all vaccine antigens, and in terms of booster response for pertussis antigens one month after booster vaccination; as well as the assessment of the safety and reactogenicity of the booster dose.

### Study participants

Participants eligible for the Primary study were healthy infants (as established by medical history and clinical examination before entering into the study) 60–90 d of age, born after a gestation period of 37–42 weeks, for whom written informed consent was obtained from the infant's parent(s) or LAR(s).

Participants eligible for the Booster study were healthy toddlers aged 12–15 months at the time of the booster vaccination who participated in the Primary study where they received 3 doses of the investigational or licensed formulation of DTPa-HBV-IPV/Hib vaccine, and for whom written informed consent was obtained.

Infants were excluded if they received any investigational or non-registered drug or vaccine within 30 d preceding the first/booster dose of the study vaccine or had a planned administration of such products during the studies. Infants who had fever (temperature ≥ 37.5°C on axillary, oral or tympanic setting, or ≥ 38.0°C on rectal setting) at the time of enrolment or who had intercurrent diphtheria, tetanus, pertussis, poliomyelitis, hepatitis B, Hib and/or pneumococcal vaccination or disease were excluded from both studies. HBV vaccination at birth and oral rotavirus vaccination were allowed at any time during the study.

### Study vaccines

Each 0.5 mL dose of DTPa-HBV-IPV/Hib contained ≥ 30 international units (IU) diphtheria toxoid, ≥ 40 IU TT, 25 *μ*g PT, 25 *μ*g FHA, 8 *μ*g PRN, 10 *μ*g recombinant HBs, 40 D-Antigen Units (DAgU) poliovirus type 1, 8 DAgU poliovirus type 2, 32 DAgU poliovirus type 3, 10 *μ*g PRP conjugated to 20–40 *μ*g TT, and 0.82 mg aluminum as salts. The 2 investigational formulations (A and B) of DTPa-HBV-IPV/Hib contained new diphtheria and tetanus antigens that differed in the process by which the detoxified antigens were adsorbed onto aluminum hydroxide, used as an adjuvant. The PRP antigen was conjugated to new TT while the other antigens were identical.

The composition of PCV13, a CRM_197_ conjugated pneumococcal vaccine, has been described previously.^8^

### Immunogenicity assessment

For each study, 2 blood samples for the analysis of antibody response were collected from all infants, before the first vaccine dose or booster and one month post-dose 3 or post-booster vaccination.

Anti-diphtheria and anti-tetanus antibodies were evaluated by an enzyme-linked immunosorbent assay (ELISA) developed in-house; seroprotection was defined as antibody concentrations ≥ 0.1 IU/mL. Anti-pertussis (anti-PT, anti-FHA and anti-PRN) antibodies were measured by ELISA (developed in-house) and seropositivity was defined as antibody concentrations above the cut-off of 5 EU/ml (ELISA units per milliliter).^9,10^ Antibodies against poliovirus types 1, 2 and 3 were determined by a virus micro-neutralization test (developed in-house),^11^ with antibody titers ≥ 8 ED_50_ (median effective dose) considered protective. Anti-PRP antibodies were measured by ELISA (developed in-house), with antibody concentrations ≥ 0.15 *µ*g/mL considered as protective.^12,13^

Regarding anti-HBs, investigations on the quality of some serology assays revealed that the anti-HBs in-house developed ELISA overestimated concentrations between 10–100 mIU/mL, while values above 100 mIU/mL were confirmed valid. Therefore, all available samples at one month after the primary vaccination for which the anti-HBs antibody concentration was between 10–100 mIU/mL by the in-house ELISA, were retested by the commercial ChemiLuminescence ImmunoAssay (CLIA; Centaur™, Siemens Healthineers) with a cut-off defining seropositivity of 6.2 mIU/mL. Anti-HBs seroprotection was redefined as in-house ELISA^14,15^ concentration above 100 mIU/mL or CLIA concentration above 10 mIU/mL. CLIA was also used as an immunogenicity assay for the Booster study. GSK Biologicals' clinical laboratories tested the samples.

### Safety assessment

Enrollment in the Primary study adopted a staggered approach and an interim safety evaluation was done to cautiously test the new formulation. During a first randomization wave, 96 infants were enrolled. A review of the safety data of those infants triggered the second enrollment wave at all study sites. Recruitment completed when at least 720 infants had been enrolled.

Solicited local (pain, redness, swelling) and general (drowsiness, irritability/fussiness, loss of appetite, fever [temperature ≥ 37.5°C on axillary]) symptoms were recorded up to 8 d after each formulation dose in the Primary study, and up to 4 d post-vaccination in the Booster study. Unsolicited AEs were recorded up to 31 d post-vaccination.

Grade 3 symptoms were defined as AEs preventing normal activity, pain upon limb movement or a spontaneously painful limb, irritability/fussiness resulting in crying that cannot be comforted, drowsiness that prevents normal activity, loss of appetite resulting in not eating at all, and grade 3 fever, as temperature > 39°C on axillary setting. All solicited local (injection site) reactions were considered causally related to vaccination. Causality of all other AEs was assessed by the investigator.

SAEs were recorded throughout the studies.

### Statistical analyses

For the Primary study, the sample size was calculated to meet the primary objectives with an overall power of at least 80% and assuming approximately 10% of non-evaluable results. A maximum of 720 (240 per group) infants were expected to be enrolled in the Primary study and therefore, potentially eligible for the Booster study, to achieve the primary objective of non-inferiority for at least one of the 2 formulations.

Descriptive statistics was used to summarize demographic characteristics. In the Primary study, the primary analyses for immunogenicity were based on the ATP-I. A second immunogenicity analysis was performed on the TVC to complement the ATP analysis. The 2 investigational formulations were assessed independently. For each new formulation, non-inferiority for immunogenicity concerning the primary endpoints was assessed sequentially according to the ranking: diphtheria, tetanus, HBs, PT, FHA, PRN and PRP. For each new formulation, a conclusion on one objective was only possible if the preceding objective with a lower rank had been demonstrated before.

The non-inferiority in terms of immune response to diphtheria, tetanus, HBs and PRP antigens was demonstrated if the ULs of the standardized asymptotic 97.5% CIs of the group difference (control group minus each investigational group [A or B]) in terms of percentage of seroprotected participants for each antigen were ≤ 10%. The non-inferiority in terms of immune response to pertussis antigens was demonstrated if the ULs of the 97.5% CI on the GMC ratio (control divided by each investigational group) was ≤ 1.5.

In both studies, descriptive analysis was done for each group, at each timepoint and for each antigen for which a serological result was available (seropositivity/seroprotection/vaccine response rates with exact 95% CIs, and GMCs or GMTs with 95% CIs).

The safety analyses were performed on the TVCs.

### Trademark

*Infanrix hexa* is a trademark of the GSK group of companies.

*Prevenar 13* is a trademark of Pfizer Inc.

On 2 March 2015 Novartis non-influenza Vaccines Business was acquired by the GSK group of companies.

## Trial registration

NCT01248884 and NCT01453998 (www.clinicaltrials.gov).

## Abbreviations

AE: adverse events
ATP: according-to-protocol
ATP-I: according-to-protocol cohort for immunogenicity
CI: confidence interval
CLIA: ChemiLuminescence ImmunoAssay
DAgU: D-Antigen Units
D_A_T_A_Pa-HBV-IPV/Hib: formulation A of DTPa-HBV-IPV/Hib
D_B_T_B_Pa-HBV-IPV/Hib: formulation B of DTPa-HBV-IPV/Hib
DTPa-HBV-IPV/Hib: combined hexavalent vaccine against diphtheria, tetanus, pertussis, hepatitis B, poliomyelitis and Hib
ED_50_: median effective dose
ELISA: enzyme-linked immunosorbent assay
FHA: filamentous hemagglutinin
GMC: geometric mean concentration
GMT: geometric mean titer
HB: hepatitis B
HBsAg: hepatitis B surface antigen
HBV: hepatitis B virus
Hib: *Haemophilus influenzae* type b
IU: international units
LAR: legally acceptable representative
Pa: acellular pertussis
PCV13: 13-valent pneumococcal conjugated vaccine
PRN: pertactin
PRP: polyribosyl-ribitol-phosphate
PT: pertussis toxoid
SAEs: serious adverse events
T: tetanus
TT: tetanus toxoid
TVC: total vaccinated cohort
UL: upper limit
